# Imaging evaluation of neoadjuvant breast cancer treatment: where do we stand?

**DOI:** 10.1007/s00330-024-10799-0

**Published:** 2024-05-16

**Authors:** Marina Álvarez-Benito

**Affiliations:** 1 Maimónides Biomedical Research Institute of Córdoba (IMIBIC) Córdoba, Córdoba, Spain; 2 Breast Cancer Unit, Department of Diagnostic Radiology, Reina Sofía University Hospital, Córdoba, Spain; 3 University of Córdoba, Córdoba, Spain

Breast cancer has become the most commonly diagnosed malignancy among women worldwide. However, there have been improvements in survival from this disease due to screening programs and the use of more effective treatments [1].

Neoadjuvant chemotherapy (NAC) is becoming the standard of care in the treatment of locally advanced breast cancer and is a treatment option for patients with early-stage breast cancer. NAC is associated with higher breast conservation rates and less need for extensive axillary lymph node dissection. The response to NAC also yields prognostic information, as pathologic complete response (pCR) after NAC is strongly associated with better long-term disease-free and overall survival, especially in patients with HER2-positive and triple-negative breast cancer [2].

Breast magnetic resonance imaging (MRI) is the most sensitive method for breast cancer detection and the most accurate imaging test for assessing tumor response to NAC. Both the American College of Radiology and the European Society of Breast Imaging recommend MRI for monitoring NAC efficacy. Evaluating the response to NAC entails comparing pre-and post-treatment MRI scans to analyze changes in maximum tumor dimensions, tumor volume, and enhancement kinetics. However, MRI can overestimate or underestimate residual tumor size after NAC. An accurate measurement of residual tumor after NAC is key to subsequent treatment planning [3–5].

The most frequently used criterion for identifying a complete imaging response is a visual assessment that shows an absence of residual enhancement at the tumor site. However, the significance of persistent T1-weighted lesions without abnormal enhancement on post-NAC MRI tests is unclear [3].

In this issue of European Radiology, the study by Goulam-Houssein et al aims to determine whether persistent T1-weighted lesions without associated abnormal enhancement on post-treatment breast MRI scans signify pCR and to evaluate their correlation with the imaging response on the MRI scans. The retrospective study was conducted on patients with breast cancer treated between January 2011 and December 2018. Patients who had a breast MRI scan pre- and post-NAC followed by surgery were included. Those with distant metastasis, no planned surgery, or pre-surgery radiation, as well as those ineligible for NAC or who had no surgical pathology reports available, were excluded [6].

The authors used the Response Evaluation Criteria in Solid Tumors (RECIST) version 1.1 to evaluate NAC response through subsequent imaging tests. Patients were deemed to have had an imaging response if they demonstrated either a complete or partial imaging response. A complete imaging response was reported when no residual enhancement was detected on post-NAC MRI studies [6, 7].

pCR was defined according to the National Surgical Adjuvant Breast and Bowel Project’s description, characterizing pCR as the absence of invasive residual disease in the breast. Univariate logistic regression was used to evaluate the association between the final pathological response and the presence of persistent T1-weighted lesions [6, 8].

Out of 319 patients, 294 met the inclusion criteria: 157 had persistent T1-weighted lesions on post-chemotherapy MRI scans, and 137 did not. A persistent T1-weighted lesion indicated the reduced likelihood of pCR (14% vs. 39%; *p* < 0.001) and imaging response (69% vs. 93%; *p* < 0.001). The ER+/HER2– breast cancer subtype was associated with significantly reduced pCR rates when compared to other molecular subtypes of invasive cancer, which is consistent with previous studies [6].

The study underscores the importance of persistent T1-weighted lesions on breast MRI scans. They are inversely related to pCR following NAC and could be a key factor in guiding post-NAC treatment decisions. Nonetheless, predicting pCR in patients with NAC prior to surgery remains a challenge [6].

It should be noted that the MRI protocol performed in this study did not include diffusion-weighted sequences, which can provide valuable early information on the effectiveness of NAC [4].

Developments and advances in breast cancer treatment will probably produce increasingly more effective drugs, increasing rates of pCR after NAC. This would allow for minimizing subsequent surgery in both the breast and axilla, with the consequent benefits for women. Some researchers even propose the possibility of omitting surgery after NAC, given the high NAC response rates in specific subgroups of responder patients in whom therapy may render surgery unnecessary. This would require methods that could predict the existence of residual tumor with a negative predictive value (NPV) of 100% [9].

Going forward, it will be necessary to advance in selecting patients who are candidates for NAC and ensuring more accurate assessments that allow for the early identification of patients who will not respond to treatment and those who may present a pCR.

A multimodal pre- and post-NAC assessment, including multiparametric MRI pulse sequences, is necessary for the correct planning of breast and axilla surgery once treatment is completed. New predictive models based on artificial intelligence and radiomic and radiogenomic image analysis combined with clinical and pathological data will play an important role in this scenario, allowing for progress toward more personalized and less aggressive breast cancer treatment [10].
